# Amino Acids and the Early Mammalian Embryo: Origin, Fate, Function and Life-Long Legacy

**DOI:** 10.3390/ijerph18189874

**Published:** 2021-09-19

**Authors:** Henry J. Leese, Paul J. McKeegan, Roger G. Sturmey

**Affiliations:** 1 Centre for Atherothrombosis and Metabolic Disease, Hull York Medical School, University of Hull, Hull HU6 7RX, UK; Roger.Sturmey@hyms.ac.uk; 2 Centre for Anatomical and Human Sciences, Hull York Medical School, University of Hull, Hull HU6 7RX, UK; Paul.McKeegan@hyms.ac.uk; 3 Division of Developmental Biology and Medicine, The University of Manchester, St Mary’s Hospital, Manchester M13 9WL, UK

**Keywords:** amino acids, preimplantation embryo, amino acid provision, nutrition, metabolism, oviduct

## Abstract

Amino acids are now recognised as having multiple cellular functions in addition to their traditional role as constituents of proteins. This is well-illustrated in the early mammalian embryo where amino acids are now known to be involved in intermediary metabolism, as energy substrates, in signal transduction, osmoregulation and as intermediaries in numerous pathways which involve nitrogen metabolism, e.g., the biosynthesis of purines, pyrimidines, creatine and glutathione. The amino acid derivative S-adenosylmethionine has emerged as a universal methylating agent with a fundamental role in epigenetic regulation. Amino acids are now added routinely to preimplantation embryo culture media. This review examines the routes by which amino acids are supplied to the early embryo, focusing on the role of the oviduct epithelium, followed by an outline of their general fate and function within the embryo. Functions specific to individual amino acids are then considered. The importance of amino acids during the preimplantation period for maternal health and that of the conceptus long term, which has come from the developmental origins of health and disease concept of David Barker, is discussed and the review concludes by considering the potential utility of amino acid profiles as diagnostic of embryo health.

## 1. Introduction

Amino acids are a zwitterionic class of compounds containing an amine and carboxyl group, with individual compounds possessing a unique side chain or R group. A large number of amino acids exist theoretically and can be synthesised chemically but, in biology, the term usually refers to the 20 specific members that form the monomeric units of proteins. In addition to these 20, there are a few biologically relevant amino acids, such as taurine and hypotaurine, which have important, if more niche, biological roles. As well as serving as the building blocks of proteins, amino acids have diverse additional functions, including energy substrates, osmolytes, antioxidants, and electron carriers.

In this review, we will examine the sources of amino acids available to the developing embryo and their physiological roles. Throughout, we will consider what remains to be discovered about the function of amino acids in early embryo development.

## 2. Origin of Amino Acids Available to Early Embryos

Amino acids are components of the fluids found within the female reproductive tract, and the addition of amino acids either singly or in combination to embryo culture media benefits embryo development. Indeed, most culture media used in clinical IVF now contain amino acids in some combination [1]. Early embryos in vivo mainly derive amino acids from these external environments. The constituents of oviduct and uterine fluids are ultimately derived from the blood but are not ‘simple’ ultrafiltrates of plasma. The epithelia lining the female tract represent the final component in a ‘supply line’ that links maternal diet at one end and embryo uptake of nutrients at the other, with further supply lines throughout pregnancy [2]. The secretions that constitute the fluid found in the oviduct are the product of this supply line. The epithelium that lines the fallopian tube is known to transport amino acids selectively from amongst a wide range of molecules, including proteins and peptides, which may be taken up and degraded to amino acids in the embryo [3]. This epithelium is also rich in ciliated cells and the proportion of the two cell types is regionally dependent, with a higher proportion of ciliated cells in the fimbriae and top end of the ampulla; a ratio which progressively shifts along the length of the oviduct such that the isthmus has a higher proportion of secretory cells [4,5]. It is widely presumed that the major role of the ciliated cells is to support gamete and embryo transport; however, these cells are also thought to contribute to the secretory function of the epithelium; indeed, ‘secretory’ cells are often referred to as ‘non-ciliated’ [6].

When considered in this way, the epithelia of the oviduct and uterus become, for a few days, the most critical maternal tissues in the establishment of a healthy pregnancy. In fulfilling this ‘gatekeeper’ role [7], female reproductive tract fluids have a key role in the ‘Developmental Origins of Health and Disease’ (DOHaD) concept [8] alongside the nature of the diet and its impact, for example, on body mass index, which in turn, can influence early embryo metabolism. This concept is revisited at the end of this review.

With regard to the timing and location of these early events, the duration of the oviduct phase is between 2–3 and 4–5 days depending on the species, by which time fertilisation and the cleavage stages of preimplantation development have been completed. The compaction stages, formation of the morula and early cavitation to yield the blastocyst coincide with transit from the oviduct into the uterus. Importantly, at these preimplantation stages, the embryo is free-living, and develops within the secretions from the epithelia. This is in contrast to later development, when the embryo implants into the wall of the uterus, at which time the developing conceptus is nourished initially by uterine histotroph before developing a vascularised support network in the form of the placenta [9,10]. The duration of preimplantation development varies considerably across mammals, from 4 days in the mouse, ~7 days in the human and ~30 days in species such as the cow and pig, where the conceptus undergoes a period of elongation prior to apposition. However, the first preimplantation stages from zygote to hatched blastocyst are directly comparable across mammals, regardless of subtle variations in the duration of specific events. During its time in the oviduct, the embryo is considered to be semi-autonomous [11], in marked contrast to the uterine phase where there is increasing maternal–conceptus interaction, which adds a radically different component to conceptus development and is out with the scope of this review. For these reasons, we have chosen to focus on the preimplantation stages of development and associated oviduct-related events, though with occasional reference to the uterus where appropriate.

## 3. Amino Acid Composition of Oviduct Fluids

Before considering what is known about the composition of oviduct luminal fluid, it is important to reflect on how we have arrived at the current state of knowledge.

There are numerous reports on the amino acid composition of oviduct fluid in a variety of species; mainly, rodents, farm animals and the human [12,13]; however, the extent to which these data reflect the true in vivo environment remains unclear, due to the methods used to retrieve fluids [7,14]. For example, the most experimentally tractable method for analysing fluid from the oviduct and uterine lumen is to retrieve samples from animals after slaughter. However, severing the blood supply risks the induction of post-mortem changes due to hypoxia, notably, lysis of the epithelial cells. This is especially a problem when there is a long time interval between slaughter and fluid collection. In the case of amino acids, a further issue arises since a characteristic of cells that carry out protein synthesis (i.e., most cells in the body, including the epithelia lining the oviduct and uterus), is the maintenance of amino acids at higher intracellular concentrations than in the surrounding extracellular fluid [15]. If cellular integrity is compromised, the contents of the cytoplasm will be released, leading to artefactually elevated amino acid concentrations in lumen contents. Despite these limitations, there have been several comparative analyses of the amino acid content of slaughterhouse-derived oviduct fluid, which offer a pragmatic opportunity to explore the nature of this critical fluid environment.

In response to the challenges of sampling oviduct fluids, [16] used the technique of acute in situ cannulation of the oviduct or uterine lumen of anaesthetized heifers. Approximately 30 min after cannulation, neat oviduct fluid appeared in the cannula, uncontaminated by cells or debris and could be collected for up to 3 h. Using this approach, [17] measured the concentrations of 19 amino acids in bovine oviduct fluids on a variety of days throughout the oestrous cycle. Nine amino acids were found at higher concentrations in oviduct fluid compared to blood plasma, supporting the proposition of active amino acid transport from the basolateral extracellular fluid into the oviduct. Interestingly, there was comparatively little variation across the oestrous cycle, suggesting that this method provides consistent data.

Nevertheless, and with the benefit of hindsight, it is important to note that aspects of the inflammatory response, notably cytokine release, are activated rapidly at sites of tissue injury such as a cannulation site, and might modify secretory rates with this and other methods for fluid retrieval. Accepting this caveat, the most abundant proteinaceous amino acids in oviduct fluid were glycine (1.98 mM) and alanine (0.6 mM); values close to those for sheep (glycine 1.5 mM; alanine 0.5 mM) reported by [18] who used a chronic method with indwelling cannulae. The same pattern, i.e., glycine and alanine as the most abundant, though with different absolute values, possibly due to different methodologies, were found for the pig [19], rabbit [20], mouse [21] and human (glycine, 0.6 mM; alanine, 0.3 mM; Tay et al., unpublished), suggesting that within the context of amino acids, the composition of oviduct fluid is to some extent conserved across several mammalian species.

Intriguingly, the amino acids present at the highest concentrations in the reproductive tract fluids of many species are taurine, and hypotaurine, an intermediate in taurine biosynthesis. These amino acids are not constituents of protein and most likely function as organic osmolytes [22] as documented in an elegant model by [23]. Of these two compounds, there are more data for taurine, with oviductal fluid concentrations relatively high in mice (6.64 mM, [21]) with lower levels in the pig (0.55 mM, [19]), rabbit (0.1 mM, [24]), cow (0.048 mM, [17]) and sheep (0.046 mM, [24]). Uterine fluid taurine concentrations are relatively high in humans (6.66 mM, [25]) and mice (3.76 mM, [21]), lower in pig (0.31 mM, [19]) and cow (0.41 mM, [17]). Hypotaurine concentrations in oviducts of a variety of animals have been reported as follows: rabbit (0.16 mM, [24]), sheep (0.27 mM, [24]), pig (0.20 mM, [24]) and cow (0.15 mM, [24]).

In the bovine oviduct, there does not appear to be active transport of taurine from plasma to the lumen with mean plasma concentrations (0.065 mM) close to those in the lumen (0.07 mM, [17]). In this context, it is interesting that (Guérin et al. [24]) reported that oviduct epithelial cell monolayers from goat, cow and pig could synthesise hypotaurine and taurine, potentially providing a further source of these amino acids in the lumen.

While there are species differences, all amino acids are selectively transported into oviduct fluids at all reproductive stages. Although it is tempting to assign significance to the active accumulation of amino acids as indicative of their special importance to the embryo, this should be done with caution for two reasons: first, new roles for amino acids in the early embryo continue to be discovered; second, many transporters in the oviduct, uterus and the embryo, are not specific for a single amino acid but may be subject to competition between one or more amino acids [26].

## 4. Proteins in Oviduct Fluid; Putative Functions and Regulation

Although not the primary focus of this review, it is important to consider the significant progress made in the study of proteins in oviduct fluid. In the context of this article, proteins are considered only as a potential source of amino acids, although it is recognised that the oviduct proteome will play many critical roles beyond amino acid supply, e.g., [13,27]. Protein concentrations in oviduct fluid are 5–10% those in serum [3,28] and the origin and transport of proteins across the oviduct and their dialogue with the gametes and embryo has become an active area of research: e.g., see reviews by [29,30,31,32]. Recent developments, well-summarised by Saint-Dizier et al. [6] have used mass spectrometry to examine the wide range of proteins present and their transport in extracellular vesicles released by the oviduct epithelial cells. Functional roles of these proteins include immune homeostasis, defense against pathogens and mediating dialogue between the maternal host and the gametes and embryo. Potential regulatory factors of these functions documented by Saint-Dizier et al. [6] include: ovulation, the proximity of the corpus luteum, insemination, presence of embryos, pregnancy, sex steroid hormones, stage of the cycle, progesterone, superovulation, oestrus synchronization, and energy balance, especially in cattle.

While most amino acids within the early embryo are likely to be derived from the external environment, it is important to acknowledge the potential role of autophagy of organelles and macromolecules in contributing to the pool of intracellular amino acids. This lysosome-mediated, pro-survival process is an area of increasing interest in gametogenesis and embryo development, as described in a valuable review by Moura et al. [33]. The capacity to carry out autophagic degradation during the preimplantation period has been shown to be obligatory in several species and is triggered by environmental stressors such as exposure of embryos to a nutrient-rich environment, as occurs in diabetes, and possibly, via Assisted Reproductive Technologies.

## 5. Transport of Amino Acids into the Early Embryo

A large number of amino acid transporters have been discovered in early embryos, some of which are specific to individual amino acids and others that transport specific groups such as branched amino acids or zwitterions. Expression of some transporters is initiated at early cleavage during Embryonic Genome Activation, but the majority begin to appear at the blastocyst stage. Prior to the onset of genome activation, the oocyte and early embryo must rely on maternal amino acid transporters.

The groundwork for this topic was laid, more than anyone, by Lon Van Winkle in a series of landmark studies that documented the various amino acid transporters in terms of their super-families and corresponding systems, throughout mouse preimplantation embryo development (see Table 1 in [34]).

Having performed this detailed characterisation, Van Winkle and others have sought to relate amino acid transporter activities to specific aspects of early embryo development; notably the initiation of blastocyst implantation, which is described below in terms of the amino acid arginine [35,36,37].

## 6. Functional Roles of Amino Acids in Preimplantation Development

There has been a revolution in thinking about the role of amino acids in cellular and molecular biology, from originally being considered predominantly as constituents of protein, they are now recognised as having multiple, overlapping functions in somatic cells, and in early embryos [38,39,40]; considered below. Aside from being utilised for protein synthesis [41,42] amino acids are also used as energy sources [43] pH buffers [44,45] and in the production of antioxidants such as glutathione [46]. Importantly, supplying amino acids to in vitro cultured embryos shifts gene expression towards a more physiological phenotype resembling that in vivo [47]. Nevertheless, it would require a much longer review to cover all aspects of amino acid function, and our focus is largely, though not exclusively, on metabolic effects.

Before discussing these metabolic functions, we feel it is important to comment (i) on the widely used distinction between ‘essential’ and ‘non-essential’ amino acids (ii) the requirement of early embryos for amino acids in culture.

### 6.1. Essential and Non-Essential Amino Acids

As pointed out by (Summers and Biggers [48] in a seminal review on early embryo culture, this terminology derives from work on the nutritional requirements of whole animals [49] and human cell lines [50], which identified 9 or 10 amino acids which had to be supplied to support animal/cell growth (‘essential’) and 7 which did not (‘non-essential’). However, it was realised that the distinction was not rigid and varied with the animal or cell type under study. Moreover, Maddy and Elvehjem, [51] showed that it was necessary to supply six amino acids in addition to Rose’s 10 ‘essential‘ ones in order for mice to grow at the same rate as on a standard (casein-based) diet. Such studies eventually led to a re-classification of amino acids into three broad categories: ‘Essential’, also termed ‘indispensable’, ‘Non-essential’, also termed ‘dispensable’ and Semi-essential, also termed ‘conditional’ (i.e., those that can be synthesised from essential amino acid precursors or become essential in some circumstances, e.g., for growth in childhood and in some disease states). However, in reality, as we have mentioned above and discussed elsewhere [39], early embryos in the female tract in all species are continually exposed to the full range of proteinaceous amino acids as well as those considered as secondary amino acids all of which, we believe, should be present in the culture medium; to supply either ‘essential’ or ‘non-essential’ represents an unphysiological situation which will restrict choice and constrain biochemical homeostasis at the cellular level.

### 6.2. The Requirement for Amino Acids of Early Embryos in Culture

The first evidence that early embryos require amino acids in culture was obtained by (Brinster, [52], who reported that two-cell mouse embryos would not develop into blastocysts in the absence of a so-called fixed nitrogen source (i.e., a nitrogen-containing compound). This could be in the form of bovine serum albumin (BSA) or a mixture of all the separate constituent amino acids in BSA. Analogous findings were obtained by Michael Kane working with Robert Foote on embryos from the rabbit [53], which, until around 1950, was the preferred animal model for studying the nutrient requirements of preimplantation embryos in culture before being replaced by the mouse [54]. The use of rabbit embryos provides a good contrast with those of the mouse since the protein content of the former is much greater than the latter; ~160 ng vs. ~25 ng at the cleavage stage and even more pronounced—440 ng vs. 24 ng—in the blastocyst. It was known that rabbit two-cell embryos would develop to the blastocyst stage in Ham’s F10 medium, a complex, cell/tissue culture medium, supplemented with BSA. Of the 4 nutrient groups in F10; amino acids, vitamins, trace elements and nucleic acid precursors, Kane found that amino acids were essential for blastocyst formation. However, these findings have tended to be neglected and it is appropriate that Kane has recently provided two fascinating articles, the first on the ‘culture of preimplantation rabbit embryos’ [55], the second, a historical note on the lessons to be learned from this research [56]. These rabbit studies have also been mentioned in some detail because they illustrate the caution that needs to be exercised in extrapolating from one animal model to another and to the human.

A large amount of further research, notably on the [57], domestic animals [58,59] and the human [60] led to amino acids being added routinely to media for culturing preimplantation embryos. As Thompson, [59] was able to state: If one had to choose the most significant “new” medium component affecting ruminant embryo development in vitro identified this decade, then the addition of pooled amino acids would rate very highly.

## 7. Metabolic Functions of Amino Acids in the Early Embryo

The major fate of amino acids in mammals is incorporation into protein, which in somatic cells is thought to account for 15–25% of total energy expenditure. The other major consumer of energy is ion pumping processes; in the embryo, this is typically the Na+, K+, ATPase, or ‘sodium pump’ enzyme, which comprises 15–25% of total energy usage. In early embryos, the energy required to operate the sodium pump only assumes major significance with the formation of the blastocoel cavity. Thus, one of us [61], in discussing the difficulty in assigning precise values to the energy costs of these two major processes during the preimplantation stages, concluded that a high proportion of the ATP generated prior to blastocyst formation must be devoted to protein synthesis. It is also more appropriate to use the term ‘protein turnover (i.e., the sum of production and breakdown) rather than ‘protein synthesis’ [62] to account for the energy cost of related processes, including protein degradation (from which there may be a small ATP yield), nucleic acid formation and the provision of amino acids. The ‘balance sheet’ from these processes is reflected, in biochemical terms, as the overall protein content of the embryo throughout development.

## 8. Protein Content of the Preimplantation Embryo

The preimplantation embryo does not ‘grow’ (i.e., exhibit a net increase in protein content) until the blastocyst stage, when there is a dramatic increase in amino acid consumption for new protein synthesis [41]. However, interestingly, a small fall in protein content, from the two-cell to morula stage, is a feature of development in the mouse [63,64], rat [65], and cow [66]. This phenomenon has perhaps been most dramatically illustrated by data for the mouse produced by Turner et al. [67], who used a scanning micro-interferometer to measure the dry mass, a good proxy measure of the protein component. The results indicated that while there was an overall loss of dry mass from the unfertilised egg to morula stage, this disguised a small fall following fertilisation and an increase at the two-cell stage, coincident with the activation of the embryonic genome, followed by the pronounced fall which precedes blastocyst formation. Intriguingly, Brinster, [63] raised the possibility that the constituent amino acids from the loss of protein might be oxidized to provide energy, which one of us [61] later calculated could theoretically fulfill the mouse embryo’s requirement for ATP for 2 days; a considerable period of developmental time. It is worth noting that if embryos used amino acids derived from the breakdown of exogenous proteins taken in by the embryo to generate ATP, this would obviate the need to expend energy in transporting individual amino acids into the cell. We are not aware of experiments to test this possibility but think it is unlikely to occur to a major extent, especially since embryos possess a full complement of amino acid transporters throughout the preimplantation period as mentioned above.

Irrespective of their origin, we can ask whether ‘surplus’ amino acids might be used as a fuel, in preference to carbohydrate and fat. The answer most likely is ‘yes’ because in the words of Frayn, [15], the enzymes for degradation and oxidation of amino acids almost exclusively have high Km (Michaelis constant) values and, thus, when amino acids are in excess, they will be degraded and oxidised in proportion to the extent of their concentration. Moreover, if there is an excess of all three fuels—carbohydrate, fat and protein—the protein will be oxidised first, since it cannot be stored in the manner of carbohydrates (as glycogen) and fats (as triglycerides). While this is most apparent at the whole body or organ levels, there is no obvious reason why it should not apply to the early embryo.

## 9. Ammonia Production

An inevitable consequence of amino acid metabolism is the need to excrete excess nitrogen, typically in the form of ammonia which is highly toxic to cells. The central factor which governs the mechanism by which ammonia is handled is the availability of water. If the supply is plentiful as in aquatic animals, ammonia, which is highly soluble in water, can be lost into the surrounding environment. By contrast, in mammals, ammonia is converted to the much less toxic product urea, mainly in the liver. 

In regards to the early embryo, (Orsi and Leese, [68] were unable to detect the formation of urea by bovine blastocysts incubated in vitro, but did demonstrate that nitrogen could be released (i) as free ammonium ions (ii) as alanine following transamination with pyruvate as proposed by Donnay and Leese, [69], (iii) as glutamine (possibly) via glutamine synthetase, and least likely, (iv) as arginine via carbamoyl phosphate synthetase. We consider that the release of ammonia into the environment at the cell surface of the embryo in vivo is unlikely to pose a problem, since the action of ciliated epithelial cells in the vicinity of the embryo and the contractile activity of the oviduct and uterine smooth muscle (myosalpinx and myometrium) will ensure mixing of the luminal fluid and minimise the build-up of unstirred layers in which ammonia might be ‘trapped’.

The production of ammonia during embryo culture in vitro is obviously dependent on the availability of amino acids, and especially of glutamine, which can break down spontaneously to ammonium when in solution. However, as a precaution against possible toxicity, glutamine is almost universally added to embryo culture media in the form of a dipeptide; usually, alanyl glutamine or glycyl glutamine, which can replace glutamine in [70,71]. Motiei et al. [72] recently reported that these dipeptides are depleted from the culture medium of viable embryos. It is presumed that they are taken up intact by the embryo and hydrolysed intracellularly to provide glutamine and the companion amino acid, though some may be broken down by extracellular, vesicle-derived peptidases released by the embryo. The details of these processes are unclear [48], especially the possible production of dipeptide breakdown products within the embryo other than the constituent amino acids. In addition, control of glutamine entry into the embryo at the level of amino acid transporters in the plasma membrane is largely lost and the embryo may have more intracellular glutamine to dispose of than is physiological. This whole area warrants further research.

## 10. One-Carbon Metabolism and DNA Methylation

Amino acid metabolism during the preimplantation stages is linked to ongoing development and later health by the processes of epigenetic modification [73]. Meaning ‘above genetics,’ epigenetic modifications modulate gene expression without altering the genetic sequence, through processes including DNA methylation, histone modification, and RNA methylation, as well as other metabolite-linked post-translational modifications [74]. These processes are intrinsically linked to metabolism and work synchronously to regulate gene expression [75]. Epigenetic changes enable cells to express a subset of the genome, allowing tissue-specific gene expression and cell specialisation.

DNA methylation, involving the addition of methyl group tags to CpG islands (CGIs), which comprise at least 55% CpG DNA, is stable and heritable [76,77,78]. It has varied effects dependent on location; thus, promoter methylation tends to repress gene expression, while intragenic methylation has gene-specific effects. Intergenic methylation is less common and less well understood [79]. Methylation is essential to cell differentiation and proliferation, with somatic cells retaining methylation patterns throughout their lifespan [80,81,82].

Epigenetic modifications are intrinsic elements of metabolism, requiring a supply of donor molecules from interlinking metabolic pathways. The primary methyl group donor for DNA and RNA methylation is the amino acid derivative S-adenosylmethionine, produced during the conversion of methionine to succinyl CoA for entry into the TCA cycle. Another key methyl donor is tetrahydrofolate, which must be converted to N5N16-methylenetetrahydrofolate by the glycine cleavage system [83]. The methionine and folate cycles are key aspects of 1-carbon metabolism, which provides donors of 1-carbon methyl moieties for a variety of processes, including DNA methylation [84]. The role of methionine is considered further in the next section on the Functions of specific amino acids.

Low-protein diets, such as those used in many rodent models of maternal diet during early development, are consequently deficient in amino acids [85]. The use of these diets has provided a valuable model system, but the resulting lack of methyl donors may partly be responsible for widespread differences in reported epigenetic modification during early development [86]. The provision of the sulphated amino acids cysteine and methionine may be particularly important. For example, cysteine is commonly low in low protein diets, and supplementation with methionine, for conversion to cysteine in the liver, is common. Cysteine deficiency reduces levels of both S-adenosylmethionine and tetrahydrofolate, and methionine deficiency has been implicated in widespread epigenetic changes in an ovine model [87]. However, supplementation with folate is required to rescue the epigenetic changes [86].

## 11. Functions of Specific Amino Acids in the Early Embryo

As mentioned above, in the context of amino acid transport, singling out individual amino acids against another is questionable since all amino acids are obviously required and new functions continue to be discovered. However, a small number of amino acids do appear to stand out as having special significance for cellular and biochemical homeostasis within the embryo. These are: arginine, leucine, glycine (with glutamate in glutathione), taurine and hypotaurine, methionine and alanine, which we discussed earlier in the context of nitrogen removal. A recent overview of the roles of amino acids in preimplantation embryos and their provision by different regions of the oviduct, has been provided by Rodríguez-Alonso et al. [13].

### 11.1. Arginine and Leucine

Arginine and leucine are considered jointly since, more than other amino acids; they are thought to have a pivotal role in regulating embryo growth and differentiation, particularly at the blastocyst stage. They are transported into the embryo through system B (0, +), which is upregulated at the blastocyst stage and preferentially triggers the mTORC1 signalling complex [88]. This is the best-known amino acid sensor [89,90] and co-ordinates the role of growth factors in differentiation and motility of the trophectoderm and its interaction with the uterus during the onset of implantation [35,36,37].

In light of such data, dietary supplementation with arginine has been proposed in order to sustain implantation and litter survival in a variety of mammals [13,37]. Arginine is also the precursor for the free radical nitric oxide produced by the enzyme nitric oxide synthase. In the mouse preimplantation embryo, NO limits oxygen consumption at the blastocyst stage [91], and may help regulate mitochondrial function to a more quiescent state, which one of us has proposed is consistent with embryo viability [92]; revised by [93] and see below). In light of these and many other studies, especially on vasodilatory effects on fetal and placental tissues, the potential value of dietary arginine supplementation in sustaining the health of mother and conceptus in later pregnancy and postnatally has been considered, though results to date are inconclusive and more data on the general issue of pregnancy nutrients and developmental programming are required [94].

Studies on somatic cells, particularly skeletal muscle, have revealed a major anabolic role for leucine as a trigger for the initiation of protein synthesis. Thus, [95] were able to state that ‘of all the Essential Amino Acids (EAAs), leucine has a particularly central role in regulating muscle protein synthesis. The provision of a small dose of leucine (3 g) to humans has been shown to provide a robust stimulation of muscle protein synthesis despite the absence of any other amino acids’. Research in this area has now reached the stage of clinical trials involving the administration of diets enriched with leucine and amino acids (especially essential amino acids) to the aged, where a fall in muscle protein synthesis capacity is closely associated with the condition sarcopenia [96]. While it is some distance, physiologically, from the intact animal to the early embryo, we consider it significant that the one amino acid consistently depleted from the culture medium by preimplantation human embryos is leucine. This was the case across three studies with different aims [97,98,99]. It is also the case that leucine is now considered alongside arginine in playing a key role in the mTOR—driven activation of the blastocyst following original work by Martin and colleagues [35,100].

### 11.2. Glycine

Glycine, along with taurine and hypotaurine (see above), are present at high concentrations in the oviduct and uterine fluids, where they act to maintain osmotic homeostasis and regulate cell volume, [101]. The use of these amino acids as organic osmolytes may be unique to the early embryo and avoids the need to use high concentrations of ions, which could otherwise disrupt cell biochemistry and electrophysiology.

### 11.3. Glycine, Taurine and Hypotaurine in Protection against Oxidative Stress

It is well-recognised that one consequence of mitochondrial respiration is the generation of Reactive Oxygen Species (ROS); notably, the free radicals, Superoxide•O_2_-perhydroxyl •O_2_H and hydroxyl •OH needed. ROS have vital roles in cells, including defence against micro-organisms and as components of signalling pathways involved in cell survival, including the induction of apoptosis, in inflammation and the immune response. However, they also cause damage to DNA, protein and lipids, and cells have evolved protective mechanisms, notable amongst which is the presence of glutathione, a tripeptide of glutamine, glycine and cysteine. Detailed discussion of the role of glutathione is beyond the scope of this review but has been expertly summarised by Menezo et al. [46] and expanded upon in a valuable review that integrates glycine into the one-carbon cycle and DNA and histone methylation [26].

### 11.4. Methionine

Methionine is a small sulphur-containing relatively hydrophobic amino acid that falls under the classification of an essential amino acid. This is notable only since many clinically used embryo sequential culture media omit the amino acids that are categorised as essential [1,102,103]. However, methionine has a number of roles in the early embryo, perhaps the most critical of which is as a precursor of S-adenosylmethionine, which is a requirement for 1-C metabolism and an essential co-factor for methyltransferase enzymes that catalyse methylation reactions. Methylation is perhaps best characterised as an epigenetic process, considered above in a metabolic context, during which methyl groups are added to nucleic acid bases, where they regulate the expression of genes. DNA methylation is the best understood of the processes which mediate the epigenetic reprogramming cycle; i.e., the erasure of epigenetic marks from primordial germ cells, their re-establishment during gametogenesis and subsequent erasure from the blastocyst stage onwards; an area well-reviewed by Huntriss et al. [104]. It has become a topic of great interest since defects in DNA methylation may be associated with the use of assisted reproductive technologies [105]. Moreover, histones and other proteins may be methylated, leading to altered function.

Proper regulation of DNA methylation is critical for early embryo development. Important work from [106] identified culture-induced stage-specific and non-stage-specific aberrant DNA methylation patterns of paternally or maternally imprinted genes and several arrays of genomic regions present in in vivo-derived bovine embryos subjected to in vitro culture in a stage-specific manner. Thus, embryos exposed to in vitro culture conditions [84] before the onset or during embryonic genome activation are more sensitive to DNA methylation marks; changes that could be further displayed in the resulting blastocysts, compared to those exposed to in vitro culture after embryonic genome activation. Furthermore, while exposure of in vivo embryos to in vitro conditions prior to embryonic genome activation favours the initiation of DNA methylation, exposing in vivo embryos to in vitro culture at the time of genome activation increases hypomethylated genomic loci in the resulting blastocysts. Studies such as these illustrate the extent to which epigenetic processes are sensitive to the conditions to which embryos are exposed. 

Within the context of methionine, Sun et al. [107] confirmed that the enzyme Methionine adenosyl transferase 2A, which converts methionine to s-adenosyl methionine, is crucial for zygotic genome activation in the mouse, and that a culture medium deficient in methionine prevented embryos from completing cavitation. More recently, Clare et al. [108] reported that methionine deficiency in a bovine oocyte model led to a reduced 1-C metabolic flux and alterations in the expression of a number of genes, including some related to fetal growth disruption. Observations such as these serve to illustrate how culture-induced epigenetic modifications that occur during preimplantation development could have a long-lasting impact on the offspring epignome and reinforce their importance of the amino acid methionine in early development.

### 11.5. Glutamine

Glutamine metabolism is prominent in proliferating somatic cells, since it contributes nitrogen atoms for de novo synthesis of the purine and pyrimidine precursors of nucleic acids (see below). Unsurprisingly, it is therefore important in early embryos; for example, it promotes the development of day 2 human embryos to blastocysts in culture [109], and its uptake is high in two and four-cell bovine embryos [110], before decreasing, in inverse proportion to glucose uptake before rising again during blastocyst expansion due to increased protein synthesis.

Two further functions of amino acids should be highlighted. The first is their role in purine and pyrimidine de novo biosynthesis, pathways which have been neglected in studies on preimplantation embryo metabolism, alongside purine and pyrimidine salvage pathways [111]. Thus, glycine with glutamine and aspartate contributes nitrogen atoms for the de novo synthesis of purines, while pyrimidine synthesis requires aspartate and glutamine. Secondly, and almost completely ignored in the early mammalian embryo is the potential role of the enzyme creatine kinase, which is present at relatively high levels in terms of biochemical activity in mouse embryos from the 2–8 cell stages before decreasing in the blastocyst [112]. Creatine kinase catalyses the breakdown of creatine phosphate to provide a rapid source of ATP which may well become critical at cytokinesis since immunocytochemical studies on two-cell embryos revealed a marked association of the enzyme with the mitotic spindle. Intriguingly, there was no obvious relationship between the gene expression of 4 subunit isoforms of creatine kinase with biochemical activity at each stage of development, providing a cautionary tale on the use of transcriptional data alone to account for physiological function.

### 11.6. The ‘Other’ Amino Acids

In Selenocysteine, known as the 21st amino acid, the sulphur in the R group of cysteine is replaced with selenium. It is genetically encoded, typically as an alternate decoding of the UGA stop codon, and found in selenium-containing proteins in most organisms, including humans [113]. Selenocysteine is a component of some glutathione peroxidase; an important part of the glutathione cycle responsible for the recycling of this key intracellular antioxidant, which is present in oocytes and early embryos. Supplementing embryo culture media with selenium has been reported to improve porcine blastocyst rates [114]. However, a specific role for selenocysteine in the mammalian oocyte or embryo has not been reported.

Other amino acids include pyrrolysine, which is similar to lysine but with a ring-containing group moiety added to the R group, and *N*-formylmethionine (fMet), similar to methionine with an oxygen-containing group added to the amino group. Both these amino acids are typically found in bacteria, while fMet is detectable in mammalian cells but far more abundant in prokaryotes, with a key role in initiating translation. Due to the prokaryotic origin of mitochondria, proteins encoded by the mitochondrial genome and translated by mitochondrial ribosomes are also initiated by *N*-formylmethionine [115,116]. *N*-formylated proteins have several reported effects in mammalian cells, such as stimulating changes in calcium signalling and chemotaxis in HL-90 promyelotic leukaemia cells [117]. However, the abundance and significance of fMet in mammalian oocytes and early embryos is currently unknown.

## 12. Amino Acids and the Early Mammalian Embryo: Life-Long Legacy

This review began by considering the ‘gatekeeper’ role of the oviduct and uterine epithelia in regulating the composition of the environments of the egg and early embryo. Following ground-breaking studies by Barker, [118] on ‘the early origins of adult disease’ convincing evidence now links the impact of these environments on the health of the embryo throughout pregnancy, perinatally, infancy and later life see companion paper in this series from Fleming et al. “Changes in Dietary Protein, Amino Acids, Folate and Other Nutrients or Toxins Regulate Embryonic and Fetal Growth and Development: Implications for Transgenerational Metabolic Disorders in Adults”.

A key question for this review is the extent to which the amino acid content in the periconceptual environment can influence the phenotype of the embryo in the short and long term. Many studies have used the ‘Low Protein Diet (LPD)’ model in the mouse, mentioned above under One-Carbon Metabolism and DNA Methylation, in which, typically, mice are mated and the females given a low protein diet solely for the duration of the preimplantation period (3–3.5 days). This changes the plasma concentration of a variety of metabolites; notably, a decrease in circulating insulin, increase in glucose and a reduction in amino acids. 

In some experiments, embryos have been transferred to the uterus of control mice fed on a standard diet. Endpoints include the proportion of embryos reaching the blastocyst stage, their implantation rate, progression through pregnancy, birth rate and post-natal development to adulthood. At each stage, a wide range of developmental, physiological, metabolic, genetic, and in the adults, psychological, markers have been measured. For example, using this approach, Eckert et al. [119] showed that LPD feeding was associated with a reduction in the content of amino acids in uterine fluid and in the blastocyst. Notable amongst the amino acids reduced were the branched-chain group; leucine, isoleucine and valine, coincident with a reduction in the growth-sensing signalling pathway mTORC1. Together, these effects were associated with compensatory changes in blastocyst growth, illustrated by proliferation of the trophectoderm and invasiveness of blastocyst outgrowth, and in later work from the same laboratory, [120], body weight gain and raised blood pressure during early postnatal life. In an excellent overview, [121] examined the influence of maternal over- as well as under-nutrition on early development, including related paternal effects and their consequences for offspring health, and were sufficiently confident to conclude:—that the evidence for periconceptional effects on lifetime health is now so compelling that it calls for new guidance on parental preparation for pregnancy, beginning before conception, to protect the health of offspring.

It is also worth reflecting on whether the supply of amino acids to the embryo is, to some extent, buffered by the fallopian tube epithelia. For example, a recent paper from Chiumia et al. [122] reported that the nature of the diet fed to dairy cows altered the composition of the fluid flushed from within the oviduct; heifers fed an ‘alpine diet’ showed a number of significant changes in metabolite composition; however, interestingly, the amino acid component remained relatively unchanged in response to diet—in support of this notion of ‘oviductal buffering’. Notably, this study reported that glycine was the most abundant amino acid within the fluid flushed from oviducts, although taurine and hypotaurine levels were not reported.

## 13. Amino Acid Depletion/Appearance (‘Turnover’); A Robust Marker of Individual Differences at the Cellular Level

There are comparatively little data on the variation in requirements for amino acids by individual oocytes and early embryos at different stages of development. Our approach to this issue has used the technique of non-invasive Amino Acid Profiling (AAP) in which individual eggs or early embryos are cultured in a small droplet of medium (4–5 µL) containing a mixture of ~20 amino acids. The droplets may be sampled periodically during embryo development for measurement of amino acid depletion and/or appearance by embryos, typically carried out using High Performance Liquid Chromatography (HPLC; see [39]). Some amino acids, such as glutamine, show a net depletion from the medium, while others, such as alanine, have a net appearance.

## 14. Diagnostic Relevance of Amino Acid Metabolism

Amino acid profiles of early embryos conducted in this way are predictive of embryo sex [123], aneuploidy [40], oxidative stress [124] and crucially, developmental capacity [97,125]. Moreover, AAP of human oocytes reflects several key features including developmental competence and hormone regimen in superovulation [126]. The method is appealing as a possible biomarker for embryo selection since it requires relatively small amounts of media (1 µL) and is highly sensitive, able to detect changes in amino acid concentrations in drops that contain single embryos or oocytes. However, translation to a clinical setting has been challenging, due to the timescales needed to perform assays, as well as the need for a number of organic solvents which are unsuitable for use in a clinical IVF setting.

Each developmental stage has a signature amino acid profile, characterised by different rates of turnover and switching between consumption and release of specific amino acids. This is true of bovine [123,127], porcine [128,129], mouse [130] and human embryos [97]. Amino acid metabolism also differs between in vitro and in vivo derived embryos [43], between male and female blastocysts and with culture medium composition. For example, removing Foetal Calf Serum (FCS) from bovine embryo culture medium reduced amino acid uptake by 30% while replacing it with Polyvinyl Alcohol reduced blastocyst rates and cell numbers [131]. FCS, as a source of amino acids, protein and many other components, is embryotrophic, but its composition is undefined, highly variable and hence unsuitable for use in clinical IVF. Instead, supplements of defined concentrations of amino acid and proteins are included in the embryo culture medium. However, their composition tends not to be shared with clinicians and researchers, making decisions on appropriate culture media that ensure that amino acid concentrations match the current understanding of best practice and physiology difficult to accomplish [132].

## 15. Amino Acid Depletion/Appearance; More Is Not Necessarily Better

**Figure 1:**
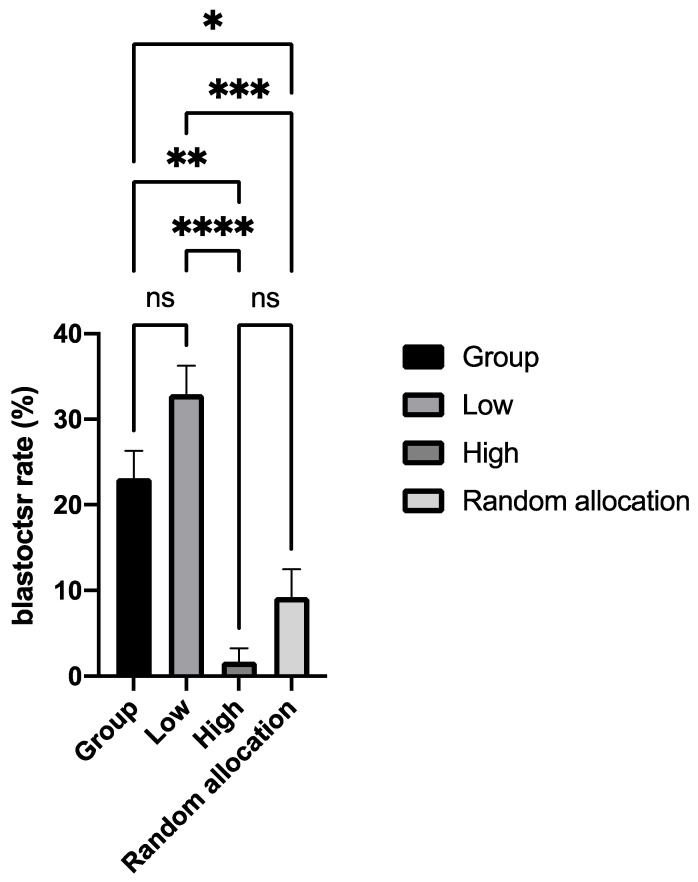
Bovine embryos that have a “lower” overall amino acid turnover produce significantly more blastocysts than to embryos with a “high” overall amino acid turnover. In order to complete this experiment, bovine embryos were cultured individually for 36h and prospectively allocated to a group based on whether their amino acid turnover was “high” or “low”. Two control groups were used; one where embryos were allocated randomly to groups after 36h individual culture and a second where embryos were maintained in group culture for the duration of the experiment. Stars indicate significant differences between groups (* *p* < 0.05; ** *p* < 0.01; *** *p* < 0.001; **** *p* < 0.0001). Redrawn from Sturmey et al. [123].

Originally, the expectation of metabolic assays was that eggs/embryos in their physiological state would give the highest amino acid depletion/appearance rates, on the basis that ‘more is better’. However, in a series of experiments conducted over a number of years, including surplus human embryos in culture [97]; cryopreserved human embryos [99]; human embryos prior to transfer [125]; aneuploid/euploid human embryos in culture [40] and bovine oocytes [126], we discovered that eggs/embryos with a normal physiology had a ‘low’ rather than ‘high’ amino acid turnover. This was well-illustrated by the data in Figure 1 which is from in vitro-produced cattle embryos (redrawn from data originally published in [123].

These data may be interpreted in terms of the ‘quiet embryo hypothesis’ [92], which proposed that healthy embryos function ‘efficiently’ in terms of the fidelity of cellular/molecular processes; they make few errors, which might lead, for example, to DNA damage [133] and therefore have a lower requirement for nutrients, especially amino acids, for repair purposes. By contrast, less efficient embryos must consume more resources, in addition to those required to sustain their normal function and thus have an ‘active’ metabolism. We later realised that the distinction between ‘quiet’ and ‘active’ embryos was too narrow and thus the hypothesis was modified to encompass the notion of an optimal range of metabolic activity within which embryos with maximum developmental potential will be located. To illustrate this, we used the principle of a ‘Goldilocks zone’, [93]; a concept widespread in biology and medicine, other areas of science and in the non-material world [134]. Such an approach could be used to define the optimum levels of amino acid turnover characteristic of a normal or abnormal egg/embryo, and in selecting embryos for transfer [135]. However, it could find much wider application, as proposed by one of us [136] in providing a model system of single cell analysis; an area of biology that is attracting much attention in light of the dramatic increase in new techniques for integrative single-cell analysis [137].

## 16. Concluding Remarks

Having been considered primarily as constituents of proteins, amino acids have emerged as the great ‘multi-taskers’ of the cellular world, involved in the numerous functions discussed in this review. In fulfilling these roles, amino acids join the traditional nutrients in early embryos: pyruvate, glucose and lactate, originally considered as energy sources, but now recognized as having multiple overlapping functions [138]. Indeed, it is conceivable that classification on biochemical grounds might be seen as too restrictive and replaced by system-based headings of the processes which occur in early embryos: maintenance, growth and repair, replication, communication with the external environment, with all such functions devoted to bringing about embryo development and calling upon macronutrients; amino acids, proteins carbohydrates, fats and their derivatives, and micronutrients, as required, to achieve this.

## Data Availability

Not applicable; no data generated.
